# Management of Diabetes and Its Complications with Banaba (*Lagerstroemia speciosa* L.) and Corosolic Acid

**DOI:** 10.1155/2012/871495

**Published:** 2012-10-02

**Authors:** Toshihiro Miura, Satoshi Takagi, Torao Ishida

**Affiliations:** ^1^Department of Clinical Nutrition, Suzuka University of Medical Science, Mie 510-0293, Japan; ^2^Department of Acupuncture and Moxibustion, Suzuka University of Medical Science, 1001-1 Kishioka, Suzuka, Mie 510-0293, Japan

## Abstract

Banaba (*Lagerstroemia speciosa* L.) extracts have been used for many years in folk medicine to treat diabetes, with the first published research study being reported in 1940. This paper summarizes the current literature regarding Banaba and its constituents. The hypoglycemic effects of Banaba have been attributed to both corosolic acid as well as ellagitannins. Studies have been conducted in various animal models, human subjects, and *in vitro* systems using water soluble Banaba leaf extracts, corosolic acid, and ellagitannins. Corosolic acid has been reported to decrease blood sugar levels within 60 min in human subjects. Corosolic acid also exhibits antihyperlipidemic and antioxidant activities. The beneficial effects of Banaba and corosolic acid with respect to various aspects of glucose and lipid metabolism appear to involve multiple mechanisms, including enhanced cellular uptake of glucose, impaired hydrolysis of sucrose and starches, decreased gluconeogenesis, and the regulation of lipid metabolism. These effects may be mediated by PPAR and other signal transduction factors. Banaba extract, corosolic acid, and other constituents may be beneficial in addressing the symptoms associated with metabolic syndrome, as well as offering other health benefits.

## 1. Introduction

**Figure 1:**
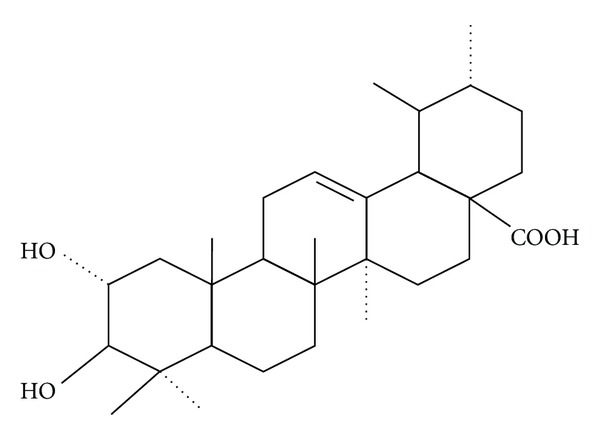
Structure of corosolic acid.

Banaba (*Lagerstroemia speciosa* L.) has been used as a folk medicine to treat diabetes in various parts of the world, primarily southeast Asia. The hypoglycemic affect of aqueous (hot water) and methanol extracts have been demonstrated in several animal models as well as a number of human studies. Most studies have focused on corosolic acid (Figure 1) which is isolated with an organic solvent from the leaves of the plant, and corosolic acid is used to standardize Banaba extracts [1, 2]. Some studies indicate that ellagitannins in water soluble fractions may be responsible for at least some of the insulin-like activity of Banaba, and the antioxidant and glucose regulatory properties of tannins in general have been reviewed by Klein et al. [3].

Corosolic acid has also been isolated from a number of other plant species including but not limited to *Vaccinium macrocarpon* (cranberry) [4], *Ugni molinae* [5], *Eriobotrya japonica* [6–10], *Perilla frutescens* [11], *Weigela subsessilis* [12, 13], *Glechoma longituba* [14], *Potentilla chinensis* [15], *Rubus biflorus* [16], and *Phlomis umbrosa* [17]. Many of these plants are native to Asia, although corosolic acid has also been isolated from European and South American plants. A discussion of the pharmacological effects of these plant species is beyond the scope of this paper.

This paper summarizes studies that have been conducted in animals, humans, and *in vitro* systems on the antihyperglycemic, antihyperlipidemic, and antioxidant activities of Banaba extracts, corosolic acid-standardized Banaba extracts, and isolated and structurally characterized corosolic acid and ellagitannins. Safety and mechanistic studies conducted to date on these various preparations are also summarized.

## 2. Human Studies

A human clinical study of Banaba was reported by Ikeda et al. [18]. A proprietary product called Banabamin in tablet form containing an aqueous extract of Banaba was used. This product also contained extracts of green tea, green coffee, and *Garcinia*. Twenty-four human subjects with mild type 2 diabetes were given three tablets three times daily. A 13.5% average decrease in blood glucose levels was reported, and no adverse effects were observed. The constituents in the product responsible for the antidiabetic effect were not determined.

Ikeda et al. also conducted a 1-year open label safety and efficacy study on 15 subjects, administering 100 mg tablets daily of a water soluble Banaba extract. The extract was not standardized, and the constituent(s) responsible for the antidiabetic effects was not determined. However, due to the low aqueous solubility of corosolic acid, the amount of corosolic acid in the water soluble preparation that was used would be expected to be low, and the hypoglycemic effects of the product may have been primarily due to water soluble ellagitannins [19].

A significant decrease (16.6%) in fasting blood glucose levels was observed in individuals with fasting blood glucose levels greater than 110 mg/dL [19]. After both 6 months and 1 year, significant improvements were observed with respect to glucose tolerance and glycated albumin following treatment with the Banaba extract. The Banaba extract did not cause hypoglycemia. No changes in hematological or biochemical characteristics and no adverse effects were observed over the 1-year course of the study.

The antidiabetic activity of a Banaba extract standardized to 1% corosolic acid in a soft gel capsule formulation has been examined [20]. Ten type 2 diabetic subjects were given 32 mg or 48 mg of the product (0.32 and 0.48 mg corosolic acid, resp.) daily for 2 weeks. A 30% decrease in blood glucose levels was reported after the 2 weeks. It is not clear whether the observed effect was due to corosolic acid, the tannin components or a combination thereof.

A 12 week lifestyle intervention study involving 56 subjects was conducted by Lieberman et al. that included the use of dietary supplements, diet and exercise [21]. The dietary supplements were taken prior to each meal, and contained 16 mg Banaba extract, 100 mg bitter melon extract, 133 mg *Gymnema* extract, 1500 mg *Garcinia cambogia* extract (60% hydroxycitric acid), 2.6 mg Bioperine (from black pepper), 10 mg wheat amylase inhibitor, 167 mcg elemental chromium, 50 mcg elemental vanadium, and 50 mg elemental magnesium. At the end of 12 weeks, the subjects had lost an average of 6.29 kg (13.8 lb) including 3.72 kg (8.2 lb) body fat as determined by a bioelectric impedance body fat analyser. Approximately, 73% of subjects completed the study. The amount of corosolic acid and ellagitannins in the Banaba extract was not reported, and it is not clear what contribution to the weight loss was provided by each constituent in the product.

In a study published by Tsuchibe et al. [22], 12 nondiabetic subjects with a baseline blood glucose level of 104 mg/dL were given a soft gel capsule daily for 2 weeks containing 10 mg corosolic acid as a Banaba extract standardized to 18% corosolic acid. A 12% decrease in fasting as well as 60 min postprandial blood glucose levels was observed after 2 weeks of administering the product. The authors also reported an average three-pound weight loss after the 2 weeks. No adverse effects were observed during or after the trial. Although this product contained a high level of corosolic acid, it is not clear if the effect was entirely due to the corosolic acid or a combination of the corosolic acid with tannin components.

In an unpublished study by Xu (“Action of helping lower blood glucose level-clinical test”, Chinese Center for Disease Control and Prevention, Beijing Hospital, 2008) using the same soft gel product containing 10 mg corosolic acid as described by Tsuchibe et al. [22], 100 subjects, with prediabetes or type 2 diabetes were enrolled. Half the subjects were given one soft gel containing the corosolic acid-standardized Banaba extract and the other half received a placebo for 30 days. Both fasting and 2 h postprandial blood glucose levels in the treated group decreased by 10% relative to the control (placebo) group. Also reported was an improvement in diabetic symptoms including a decrease in thirst, drowsiness, and hunger. Furthermore, no adverse effects were observed, with no changes in blood pressure, liver or kidney function, blood cell count, or hemoglobin.

Fukushima et al. published a study involving 31 subjects in a double-blind cross-over design that were given a capsule containing 10 mg corosolic acid or a placebo 5 min before a 75 g oral glucose tolerance test. Blood glucose levels were measured at 30 min intervals for 2 h. The authors reported that corosolic acid treatment resulted in lower blood glucose levels from 60 to 120 min compared with controls, the difference being statistically significant (*P* < 0.05) at the 90 min time point. According to the authors, the corosolic acid that was used was 99% pure, thus indicating that the blood sugar lowering effect was specifically due to the corosolic acid [23].

A single report has suggested that corosolic acid may have been involved in nephrotoxicity and lactic acidosis in a diabetic patient with impaired kidney function who was also taking diclofenac for joint pain [24]. Diclofenac is a nonsteroidal anti-inflammatory drug, and this class of drugs is known to cause renal damage and failure. The role of corosolic acid, if any, is not clear. The use of a drug known for its nephrotoxicity in conjunction with impaired kidney function readily explains the resulting kidney failure. The ability of corosolic acid to inhibit gluconeogenesis could favor lactic acid production. If corosolic acid impaired the metabolism of diclofenac, it could theoretically have exacerbated the known nephrotoxicity of the drug. No evidence was provided to specifically demonstrate this possible effect, and no controlled clinical studies have reported nephrotoxicity in diabetic subjects receiving corosolic acid.

The above clinical studies demonstrate that Banaba extract, Banaba extract standardized to corosolic acid and corosolic acid, itself decrease fasting as well as postprandial blood glucose levels in humans. A decrease in blood glucose levels has been observed within 2 h of dosing, and the decrease is typically in the range of 10–15%, although a decrease of 30% has been reported. No adverse effects have been observed or reported in any studies involving human subjects receiving Banaba, including one study involving 15 subjects who were given Banaba extract daily for up to 1 year. 

## 3. Animal Studies

The first published research on the hypoglycemic, insulin-like activity of Banaba was reported by Garcia [25, 26]. An aqueous extract equivalent to 1-2 g of dried leaves per kg body weight given orally to rabbits lowered blood sugar for 4–6 h.

Various animal studies have subsequently shown that Banaba extracts of unknown composition, Banaba extracts standardized to corosolic acid, and highly purified corosolic acid exert beneficial effects with respect to blood glucose and lipid regulation. Kakuda et al. fed genetically diabetic (KK-AY) mice diets containing 5% of a hot water extract and 2% of a methanol extract of Banaba leaves for 5 weeks. The elevation of blood glucose levels was significantly suppressed by feeding either of the two extracts. The levels of serum insulin, plasma total cholesterol, and amount of urinary glucose were all lowered by feeding the extracts, with somewhat greater activity with the water extract that was given at a higher concentration. The specific constituents in the extracts responsible for these effects were not determined [27].

A hot water extract of Banaba leaves suppressed blood glucose elevation following starch administration but not after glucose administration to rats [28]. These investigators demonstrated that the extract inhibited the activities of various hydrolytic enzymes including *α*-amylase, glucoamylase, isomaltase, maltase, and sucrase. The constituents in banaba responsible for these enzyme inhibitory activities were not determined.

Yamaguchi et al. fed 0.072% corosolic acid in the diet to spontaneously hypertensive rats for 14 weeks. The investigators reported a significant decrease in blood pressure, serum-free fatty acids, and oxidative stress markers relative to the diet containing no corosolic acid. However, they observed no effect of the corosolic acid in the diet on body weight gain or blood glucose levels [29]. A study by Matsuura et al. examined the abilities of various teas (aqueous decoctions), including from Banaba leaves, to suppress the elevation of blood glucose in rats from continuous intragastric infusion of sucrose or maltose. Banaba had no significant effect on glucose levels. The composition of the Banaba extract was not reported. The reason for the lack of effect of Banaba on blood glucose levels in these two rat studies is not known. These results are in sharp contrast to the results presented below that primarily involved mouse studies as well as streptozotocin-induced diabetic rats, and the human studies reported above [30].

In a study involving genetically diabetic mice given an extract of Banaba (0.8 mg/kg body weight) for 12 weeks orally, no effect was observed on fasting blood sugar levels, hemoglobin A1C content, body weight or insulin levels [31]. However, kidney glucose-6-phosphatase activity was significantly lower than in control animals. The most likely explanation for the poor antidiabetic effect is that the dose of the extract was too low. In addition, the extract had not been standardized or analysed with respect to potential active constituents.

Yamada et al. examined the effects of feeding mice a high fat diet for 9 weeks with and without 0.023% corosolic acid (not a standardized extract of Banaba). Corosolic acid treatment reduced fasting plasma levels of glucose, insulin and triglycerides by 23%, 41%, and 22%, respectively. A 10% decrease in body weight and a 15% loss in fat total mass were also observed relative to control animals. In addition, the corosolic acid in the diet increased the expression of peroxisome proliferator-activated receptor-alpha (PPAR-*α*) in the liver and PPAR-*γ* in white adipose tissues, thus providing a mechanistic explanation for the loss in body weight and the decrease in hepatic steatosis in these mice. These results indicate that corosolic acid may be beneficial in addressing various aspects of the metabolic syndrome that consists of hyperlipidemia, obesity, hypertension, and insulin resistance. The aspects of metabolic syndrome which may be addressed by corosolic acid include obesity, insulin resistance, and hypertriglyceridemia and hypercholesterolemia [32].

Yamada et al. also investigated the mechanism of action of corosolic acid on gluconeogenesis in rat liver using perfused livers and isolated hepatocytes. Corosolic acid (20–100 *μ*m) in a dose-dependent manner decreased gluconeogenesis by increasing production of fructose-2,6 diphosphate by lowering cyclic AMP levels and inhibiting protein kinase A activity. In addition, corosolic acid increased glucokinase activity without affecting glucose-6-phosphatase activity, suggesting an increase in glycolysis. The results provide additional mechanistic information regarding the antidiabetic actions of corosolic acid [33].

Deocaris et al. administered various Banaba leaf extracts of unknown and unstandardized composition prepared with 80% ethanol, subcutaneously to alloxan-induced diabetic mice. Banaba leaf extract had minimal effects on blood glucose levels, but when combined with insulin, the activity was synergistically enhanced. Gamma irradiation of Banaba leaves led to extracts with higher hypoglycemic activity when mixed with insulin than unirradiated extracts. Irradiation appeared to lead to improved extraction efficiency of the active component (s) [34].

A study was conducted involving genetically diabetic (db/db) mice fed with a diet supplemented with a water soluble extract of Banaba at a 0.5% concentration [35]. At the end of 12 weeks, animals receiving the Banaba extract exhibited significantly reduced blood glucose, insulin, triglycerides, and hemoglobin A1C levels. Furthermore, increased expressions of liver PPAR-*α* mRNA and lipoprotein lipase (LPL) mRNA as well as adipose tissue PPAR-*γ* mRNA were observed. The results suggest that the Banaba extract increased insulin sensitivity and blood sugar regulation by regulating PPAR-mediated lipid metabolism. However, the identity of the responsible factor(s) in Banaba was not determined.

Miura et al. conducted several studies in genetically diabetic (KK-AY) mice to which corosolic acid was administered. At a single dose of 10 mg/kg, corosolic acid significantly reduced blood sugar levels. This effect was shown to be associated with an increase in the muscle glucose transporter (GLUT4) [36]. In a subsequent study, they showed that a single dose of 2 mg/kg corosolic acid reduced blood sugar levels for up to 2 weeks, supporting the hypothesis that corosolic acid improves glucose metabolism by reducing insulin resistance [37].

In a study in mice, Takagi et al. demonstrated that an oral dose of 10 mg/kg corosolic acid suspended in water inhibited the intestinal hydrolysis of sucrose but not maltose or lactose, thereby at least in part facilitating the lowering of blood glucose levels since sucrose is a disaccharide composed of glucose plus fructose. In this study, a sugar solution was administered 30 min orally after the corosolic acid, and blood samples were drawn at 30, 60, and 120 min [38]. These results agree with the observations of Suzuki et al. who showed that an extract of Banaba leaves inhibited sucrase activity and exerted hypoglycemic effects through multiple mechanisms [27].

Takagi et al. have shown that when genetically diabetic (KK-Ay) mice are fed a high cholesterol diet with and without 0.023% corosolic acid for 10 weeks, the corosolic acid significantly decreased blood cholesterol and liver cholesterol content by 32% and 46%, respectively. Furthermore, the diabetic mice were given corosolic acid (10 mg/kg body weight) orally in water followed by the oral administration of a high cholesterol cocktail 30 min later. Corosolic acid significantly inhibited mean blood cholesterol levels 4 h after administration of the cholesterol relative to control animals. The effect was believed to be due to inhibition of cholesterol absorption via inhibition of the enzyme cholesterol acyltransferase [39].

Several studies have examined the effects of aqueous Banaba extracts on streptozotocin-induced diabetic rats. In none of these studies were the active constituents determined. A hot water (75–90°C) extract of Banaba leaves was shown to depress the elevated blood glucose levels in streptozotocin-diabetic rats by about 43%, while increasing the activity of glucose-6-phosphate dehydrogenase as well as glutathione content, and decreasing the activity of the gluconeogenic enzymes glucose-6-phosphatase and fructose-1,6-diphosphatase [40]. The dose of the extract used and the duration of the study were not reported. The results suggest that the hypoglycemic activity occurs through suppression of gluconeogenesis and stimulation of glucose oxidation via the pentose phosphate pathway.

Thuppia et al. prepared an extract of Banaba leaves by boiling for 2 h which was subsequently freeze-dried. The extract was administered orally at doses up to 2000 mg/kg body weight for 12 days to streptozotocin-diabetic rats. A dose of 1000 mg/kg decreased fasting blood glucose levels by approximately 43% on day 12, which returned to pretreatment levels by day 3 after cessation of treatment. The Banaba extract produced no changes in blood glucose levels in nondiabetic rats. The high doses of the extract needed to produce a hypoglycemic effect may be reflected in the extraction procedure where boiling may have destroyed some of the active constituents [41].

An aqueous extract (150 mg/kg body weight) given to streptozotocin-induced diabetic mice for up to 15 days significantly decreased not only blood glucose levels but also exhibited a potent antioxidant effect [42]. Administration of the extract reduced streptozotocin-generated reactive oxygen species (superoxide anion, hydrogen peroxide, and nitric acid) by upregulating superoxide dismutase, catalase and glutathione-S-transferase activities as well as reduced glutathione levels.

Administration of a spray-dried extract of Banaba (100 mg/kg body weight) for 28 days by gavage to alloxan-induced diabetic mice resulted in significantly lower blood and urine glucose levels [43]. In addition, lower food and fluid intakes as well as lower body weights were observed in response to the Banaba extract.

Oleanolic acid is a pentacyclic terpene acid that is structurally related to corosolic acid, has been isolated from Banaba leaves, and exhibits *α*-glucosidase activity [7]. Oleanolic acid and insulin were shown to decrease blood glucose levels in control and streptozotocin-induced diabetic rats given a glucose load after an 18 h fast [44]. Furthermore, daily treatment for 5 weeks with oleanolic acid significantly decreased blood glucose levels in diabetic animals with concomitant restoration of hepatic and muscle glycogen stores to near normal levels and in combination with insulin provided even greater antihyperglycemic activity. Oleanolic acid may act via a mechanism distinct from insulin including its *α*-glucosidase activity, and the two may exert a synergistic effect in the regulation of hyperglycemia.

The anti-inflammatory activity of various pentacyclic triterpene acids including corosolic acid was assessed by Aguirre et al. *in vivo* using a mouse ear assay, using arachidonic acid and 12-O-tetradecanoylphorbol-13 acetate as the inflammation-inducing agents. Corosolic acid was effective against both inflammatory agents [5].

In summary, various studies have demonstrated that Banaba extracts as well as corosolic acid significantly decrease blood glucose levels in genetic as well as streptozotocin- and alloxan-induced diabetic animals. Better responses appear to occur in mice compared with rats. In these studies, corosolic acid has also been shown to improve insulin sensitivity, increase cellular uptake of glucose, decrease serum triglycerides and cholesterol, facilitate weight loss, and improve oxidative stress markers without production of adverse or toxic effects. In addition, the animal studies suggest that Banaba extracts and corosolic acid may have additional health-enhancing benefits that extend beyond the effects observed to date in human subjects. The animal studies also support the safety findings reported in human studies and have provided extensive information on the mechanisms of action of Banaba extract and corosolic acid. 

## 4. *In Vitro* Studies

The antioxidant and free radical scavenging activities of Banaba were demonstrated for an aqueous extract in *in vitro* free radical generating systems in a concentration dependent manner [45]. The Banaba extract was shown to have potent radical scavenging activity on 1,1-diphenyl-2-picrylhydrazyl (DPPH) radical and superoxide radicals generated by a hypoxanthine-xanthine oxidase system. The extract also inhibited lipid peroxidation in a rat liver homogenate system. The tannin content of the extract was about 37% of dry weight.

Liu et al. demonstrated that both water and methanol extracts of Banaba stimulated glucose uptake by 3T3 adipocytes. The extracts also inhibited adipocyte differentiation induced by insulin. The active components in these Banaba extracts were not identified [46].

Tanaka et al. isolated and structurally characterized various ellagitannins from the fruit and leaves of Banaba. Lagerstannins A and B together with five known tannins including lagerstroemin were isolated from the fruit, while lagerstannin C was isolated and characterized from Banaba leaves. The three lagerstannins possess a gluconic acid core which is rarely found in the plants [47].

Three ellagitannins, lagerstroemin, flosin B, and reginin were extracted from Banaba and shown to increase glucose uptake by isolated rat adipocytes [48]. Lagerstroemin exhibited glucose transport stimulation with a 50% effective concentration (EC_50_) of 80 *μ*m, with a maximum effect of approximately 54% of that of insulin. However, it is doubtful that this concentration of lagerstroemin can be achieved *in vivo* following oral administration of the doses commonly used for Banaba extracts. Furthermore, tannic acid, which is commercially available and widely distributed in plants, has been shown to exhibit glucose transport activity with an EC_50_ of 17 *μ*m, approximately five times more potent than lagerstroemin [49]. Thus, it is not plausible to ascribe the glucose regulatory activity of Banaba extracts specifically to lagerstroemin.

Subsequent studies with lagerstroemin demonstrated that it exhibited insulin-like activities including increasing glucose uptake and decreasing isoproterenol-induced glycerol release in rat adipocytes, and increasing extracellular signal-related kinase (Erk) activity in Chinese hamster ovary cells [50]. It should be noted that these studies were conducted *in vitro*, and similar activities have not been demonstrated in animal or human systems.

More recently, Bai et al. have isolated and structurally characterized seven ellagitannins and four methyl ellagic acid derivatives from Banaba leaves. A number of polyphenolic compounds including corosolic acid and quercetin were also isolated. The ellagitannins all exhibited the ability to stimulate insulin-like glucose uptake as well as to inhibit adipocyte differentiation in 3T3-L1 adipocyte cells in culture. Furthermore, the methyl ellagic acid derivatives exhibited inhibitory activity with respect to glucose transport. These studies clearly demonstrate the antihyperglycemic activity of well characterized ellagic acid derivatives from Banaba, show that this activity is not restricted to a single compound, and indicate that multiple mechanisms of action are involved [51].

Hosoyama et al. conducted a quantitative analysis of an *α*-amylase inhibitor in aqueous Banaba leaf extracts. The extracts were hydrolysed with hydrochloric acid, extracted with an organic solvent and subjected to high performance liquid chromatography. Using bioassay-guided analysis of various fractions, the polyphenolic valoneic acid lactone was isolated and identified as an *α*-amylase inhibitor with an IC_50_ of approximately 108 *μ*g/mL. However, no standard *α*-amylase inhibitor was used. The authors measured the valoneic acid content of eight Banaba leaf decoctions, and showed that the *α*-amylase inhibiting activities were correlated with the content of this polyphenolic acid. The *α*-amylase inhibiting activity of corosolic acid was not determined, and may not have been present in large amounts since water was used to make the initial leaf extracts [52].

The corosolic acid content of Banaba leaves, Banaba methanol extracts and various commercial dosage forms have been determined using high performance liquid chromatography (HPLC) as well as high performance thin layer chromatography (HPTLC) by Vijaykumar et al. [53]. Several Banaba leaf samples were shown to contain 0.31–0.38 mg corosolic acid/100 mg, while methanol extracts of leaves contained up to 11.3 mg/100 mg.

Using a Chinese hamster ovary cell system, Shi et al. demonstrated that corosolic acid stimulated glucose uptake via enhancing insulin receptor phosphorylation. Furthermore, corosolic acid inhibited several diabetes-related nonreceptor protein tyrosine phosphatase enzymes. These studies provide supporting and mechanistic information regarding the ability of corosolic acid to exert a hypoglycemic effect [54]. Although these studies showed the phosphorylation of the insulin receptor directly, other studies could not repeat this result.

The ability of an aqueous Banaba extract to block the activation of nuclear-factor- (NF-) *κ*B by tumor necrosis factor (TNF) in a dose- and time-dependent manner was demonstrated using the cardiomyocyte cell line H9c2 [55]. The authors suggested that this anti-inflammatory action might explain the ability of Banaba extract to inhibit diabetes-induced cardiomyocyte hypertrophy. This study provides mechanistic insight into the anti-inflammatory activity demonstrated by corosolic acid and other pentacyclic terpene acids in mice [5].

Six pentacyclic triterpene acids (oleanolic acid, arjunolic acid, asiatic acid, maslinic acid, corosolic acid, and 23-hydroxyursolic acid) were isolated from Banaba leaves by ethyl acetate extraction [7], and their abilities to inhibit *α*-amylase and *α*-glucosidase activities were determined. Corosolic acid exhibited the greatest inhibitory activity against *α*-glucosidase with an IC_50_ of 3.53 *μ*g/mL, while all of the six pentacyclic triterpenes exhibited weak or no inhibitory activity against *α*-amylase. These results provide additional information regarding the mechanisms of action of Banaba with respect to its antidiabetic activity.

An interesting study regarding the anabolic effects of corosolic acid on osteoblastic bone formation was reported by Shim et al. [56]. Concentrations up to 5 *μ*m corosolic acid significantly stimulated differentiation of mouse osteoblasts. This effect was shown to be mediated by activation of mitogen activated protein kinase (MAPK), NF-*κ*B and activator protein-1. These results suggest that corosolic acid may be useful in conjunction with bone diseases such as osteoporosis and periodontitis.

In summary, various *in vitro* studies involving cell-free systems and cell cultures have demonstrated antioxidant and osteoblastic activities of Banaba extracts and corosolic acid. In addition, various ellagitannins and methyl ellagic acid derivatives have also been isolated from Banaba and have been shown to exhibit antihyperglycemic activity. Furthermore, much information and insight regarding the mechanisms of action of Banaba extracts, corosolic acid and ellagitannins have also been obtained using these *in vitro* systems. 

## 5. Conclusions

A growing body of evidence involving animal and human studies as well as *in vitro* systems indicates that Banaba leaf extracts exert antidiabetic and antiobesity effects. There is strong evidence to indicate that corosolic acid as well as ellagitannins is responsible for these effects. Other polycyclic terpene acids such as oleanolic acid and valoneic acid may also contribute to the antihyperglycemic effects. With the development of techniques to purify various components of Banaba, studies are now being conducted with more highly purified and structurally characterized materials. As a consequence, information is being obtained regarding the specific effects of the various constituents, particularly with respect to corosolic acid.

A number of studies in animals and human subjects using highly purified corosolic acid and corosolic acid-standardized preparations indicate that this component of Banaba exhibits properties that are beneficial in addressing various factors involved in glucose regulation and metabolism, including the enhanced cellular uptake of glucose, improved insulin sensitivity, decreased gluconeogenesis, and inhibited intestinal hydrolysis of sucrose, thereby lowering blood glucose levels. Furthermore, decreased serum cholesterol and triglycerides have been observed in response to corosolic acid.

Based on the studies conducted to date, no adverse effects have been reported in animals using either corosolic acid or standardized Banaba extracts, nor have adverse events been observed or reported in controlled human clinical studies. However, no animal studies have been designed specifically to assess toxicity or LD_50_ values for corosolic acid or Banaba extracts standardized to specific concentrations of corosolic acid.

The above studies indicate that corosolic acid and corosolic acid standardized Banaba extracts may be beneficial in addressing issues associated with elevated blood sugar levels and obesity. Furthermore, corosolic acid exhibits anti-inflammatory and antihyperlipidemic, antiviral effects. Standardized Banaba extracts, corosolic acid, and/or ellagitannins in combination with other ingredients may be useful in dealing with symptoms associated with metabolic syndrome. Corosolic acid and standardized Banaba extracts may also be highly effective either as stand-alone products or in combination with other natural products possessing hypoglycemic, antihyperlipidemic, and appetite suppressant activities.

Additional human efficacy and safety studies are warranted, particularly studies assessing the dose- and time-dependent effects of corosolic acid or corosolic acid-standardized Banaba extracts and ellagitannins alone or in combination with other ingredients on blood lipids (triglyceride and cholesterol), insulin and glucose levels as well as weight loss, and weight management. Investigations are needed to clearly define and understand the roles and importance of corosolic acid and related pentacyclic terpene acids relative to the ellagitannins present in Banaba [57]. Finally, additional acute and subchronic animal safety studies are needed.
